# Factors Explaining Responses to Influenza and COVID-19 Vaccination Among Nurses in Israel

**DOI:** 10.3390/vaccines13050454

**Published:** 2025-04-24

**Authors:** Ola Ali-Saleh

**Affiliations:** Department of Nursing, The Max Stern Yezreel Valley College, Emek Yezreel 1930600, Israel; olaa@yvc.ac.il

**Keywords:** influenza, COVID-19, vaccine, nurses, Israel, health belief model, theory of reasoned action

## Abstract

**Background/Objectives**: During the COVID-19 pandemic, influenza vaccination compliance among nurses in Israel was significantly lower than in previous years. This study sought to evaluate factors associated with vaccination compliance. **Methods**: An online cross-sectional survey conducted in March-April 2022 among 386 Israeli nurses examined perceived disease threat, vaccination barriers, perceived vaccine benefits, attitudes, and subjective norms/social influences. **Results**: During the 2021/2022 winter season, the vaccination rate for COVID-19 was higher than for influenza (68.4% vs. 61.9%). For both, vaccination compliance was positively associated with perceived susceptibility and severity, perceived benefits, and supporting attitude and negatively associated with barriers. The odds for COVID-19 vaccination were higher among older (*OR* = 1.04, 95% *CI* = 1.02, 1.07, *p* < 0.001) and more experienced nurses (age and years of experience, *r* = 0.89, *p* < 0.001). For both, perceived susceptibility and severity were higher among female nurses (influenza *M* = 3.29 *SD* = 0.88; COVID-19 *M* = 3.65 *SD* = 0.83) than male nurses (influenza *M* = 3.03 *SD* = 0.90; COVID-19 *M* = 3.32 *SD* = 0.83). A model assessing the associations between COVID-19-related variables and influenza vaccination compliance found that higher perceived susceptibility and severity regarding COVID-19, lower perceived barriers to COVID-19 vaccination, and more supportive attitudes toward COVID-19 vaccination were related to a greater likelihood of influenza vaccination compliance. **Conclusions**: Perceived susceptibility, perceived severity, and attitudes made a significantly greater contribution to influenza vaccination than to COVID-19 vaccination, whereas perceived benefits made a significantly greater contribution to COVID-19 vaccination than to influenza vaccination.

## 1. Introduction

Influenza is a viral disease that occurs mainly during the winter and causes severe respiratory symptoms, including fever, contagious cough, and nose, throat, and lung infections. The virus is easily spreadable, and affected individuals often miss up to seven days of work. Therefore, to prevent the symptoms, shorten the length of the illness, and avoid complications for individuals at high risk, the World Health Organization (WHO) recommends annual influenza vaccination [1]. Within ten days to two weeks after vaccination, the body develops specific antibodies that act as a defense mechanism, reducing the probability of contracting influenza upon exposure to the virus [2]. Vaccination is particularly beneficial to vulnerable population groups such as older adults, young children, pregnant women, and immunocompromised individuals. Healthcare workers (HCWs) constitute another group liable to be infected by the influenza virus. High-caliber HCWs play an important role in public medical services by caring for and treating patients, yet they must exercise caution to avoid transmitting the virus to susceptible population groups [3,4,5].

At the outbreak of the COVID-19 pandemic and before the COVID-19 vaccine became readily available, the healthcare system adopted the logic of vaccinating a high percentage of the population, and especially healthcare workers, against circulating strains of influenza. One reason for this policy was to differentiate between influenza and COVID-19 infections due to the similarity of their symptoms [5,6].

Every year, the WHO publishes a forecast of influenza strains expected for the coming winter in Northern Hemisphere countries, as determined by virus prevalence in Southern Hemisphere countries. Based on this forecast, pharmaceutical companies manufacture a vaccine targeting the specific [dominant] strains for the coming season [7].

The outbreak of the coronavirus disease pandemic in 2019 (COVID-19) placed an immense load on the health system due to the extremely contagious nature and severity of the disease [8]. In March 2020, the WHO declared the COVID-19 outbreak a pandemic with a high mortality rate, reaching over 1 million deaths worldwide by October 2020 [9]. As a consequence, immediate protective equipment, including face masks, gloves, and gowns, was dispersed to healthcare workers [10]. In December 2020, Israel took an important step to prevent COVID-19 infection and began administering the COVID-19 vaccine (BNT162b2, developed by BioNTech and Pfizer). Two doses of the vaccine were administered, with a three-week interval between them [11].

By 2022, overall uncertainty worldwide concerning control of the new infectious virus drew attention away from annual anti-influenza vaccination. Hence, reports indicated lower rates of influenza vaccination among healthcare workers in Israel in 2022. The most significant decline in the influenza vaccination rate was observed among nurses (a decrease of 30% compared with the 2020–2021 season) [1,12]. This decline occurred despite research showing that vaccinating healthcare workers against influenza helps combat the spread of the disease and reduces morbidity rates among high-risk populations, such as hospitalized patients, long-term geriatric care residents, young children, pregnant women, elderly people, and immunocompromised individuals [13]. Additional advantages of influenza vaccination include protecting vulnerable populations from complications such as pneumonia and morbidity, which is an utmost priority, along with minimizing absences among healthcare workers who come in contact with ill patients and therefore may be exposed to the virus [14,15].

Although influenza viruses pose health risks that could be avoided by vaccination, the rate of vaccination compliance, even among healthcare workers, was lower than expected [14,16]. According to several studies reporting influenza vaccine coverage among healthcare workers (nurses) in Italian hospitals during the 2020–2021 season, vaccination rates were lower than the 75% target proposed by the Italian Ministry of Health [3,4].

The current study assesses the rate of influenza vaccination among nurses. These HCWs are the first responders to come in contact with the public. The present study aims to examine the factors explaining compliance with influenza and COVID-19 vaccinations among nurses. Understanding compliance with vaccination against two different viruses that broke out simultaneously is important for future scenarios. Another purpose of this study is to assess the relationship between variables associated with COVID-19 and compliance with the influenza vaccine. This important and unique research can support our understanding of the factors related to responding to different vaccines during a pandemic.

## 2. Materials and Methods

In the present research, an online cross-sectional survey was conducted in March-April 2022 among nurses in Israel. The questionnaire was distributed via social networks such as WhatsApp and Facebook. Participants were asked to forward the questionnaire link to their nursing colleagues who met the criteria for this study (snowball sampling technique). The inclusion criteria were professional nurses actively working in the Israeli health system. Nurses who are unemployed and HCWs who are not nurses were excluded. This study was conducted in accordance with the Declaration of Helsinki and approved by the Emek Yezreel Academic College Ethics committee (Approval No. YVC EMEK 2022-42). Participants gave their consent by answering the survey.

### 2.1. Questionnaire Design

The research questionnaire consisted of three parts and was based on validated questionnaires used in previous studies [17,18]. The questions were adapted for the purpose of the present study.

#### 2.1.1. Demographic Details

Age, gender, religion (Jewish, Muslim, Druze, or Christian), degree of religiosity (secular, traditional, religious, or very religious), marital status, number of children, education and professional experience, and workplace details were collected.

#### 2.1.2. Subjective Health Assessment

The following information was collected: subjective health assessment rated as 1—not good to 5—excellent; chronic disease: yes/no; taking medication to treat chronic diseases: yes/no.

#### 2.1.3. Statements About COVID-19 Vaccine and Influenza Vaccine

The questionnaire included statements regarding perceived disease threat, barriers to vaccination, perceived benefits of the vaccine, and incentives for action. These statements were based on the Health Belief Model (HBM) [19,20].

The perceived threat of influenza/COVID-19 variable consisted of two parts: (1) Perceived susceptibility to influenza included three items. Sample item: “I think I am at higher personal risk of getting influenza/COVID-19”. (2) Perceived severity of the disease included two items. Sample item: “Influenza/COVID-19 is dangerous for my patients” (influenza α = 0.77, and COVID-19 α = 0.73).

Perceived benefits of the vaccine included four items. Sample item: “Vaccination against influenza/COVID-19 reduces the risk of infecting patients” (influenza α = 0.91, and COVID-19 α = 0.90).

Perceived barriers to vaccination included six items. Sample item: “I’m afraid of long-term side effects after the vaccination” (influenza α = 0.73, and COVID-19 α = 0.64).

The questionnaire also included statements examining attitudes, subjective norms, and incentives for action, which were composed according to the Theory of Reasoned Action (TRA) [21,22]. Subjective norms and attitudes regarding compliance with influenza vaccination as well as social influences and attitudes were assessed with the help of questionnaires by Fishbein and Ajzen [21,22] adapted to the present study.

Attitudes about the influenza/COVID-19 vaccine were evaluated using five items. Sample item: “The influenza/COVID-19 vaccine should be mandatory for healthcare workers” (influenza α = 0.89, and COVID-19 α = 0.91).

Social influences/subjective norms were assessed by two items. Sample item: “Most of my co-workers believe it is important to get vaccinated against influenza/COVID-19”.

### 2.2. Participants

The participants in this study were 386 Israeli nurses, 22–62 years old, with a mean age of about 37 years, who were mostly female (about 73%) (Table 1). Most were married or in an intimate relationship (about 74%) and had children (about 69%). Close to half were Muslim, about 30% were Jewish, and the rest were Christian or Druze. About 37% were secular, and the others were partly religious (about 40%) or religious (about 23%). Most nurses had an academic degree, with up to 41 years in the profession, and a mean of about 11 years. Most worked in hospitals (about 61%), while others worked in the community, e.g., clinics and private physician offices (about 18%) or in nursing homes (about 21%). Most reported very good to excellent health, with no chronic illnesses or permanent medication.

### 2.3. Vaccination

Influenza was defined dichotomously as being vaccinated in both 2020/2021 and 2021/2022 (1) versus being vaccinated in one or none of these two years (0).

COVID-19 was defined dichotomously as taking three doses of the vaccination or being sick with COVID and taking two doses of the vaccination (1) versus less than that (0).

### 2.4. Data Analysis

Data were analyzed with SPSS version 28 (IBM, New York, NY, USA). Descriptive statistics were used with the demographic variables and the study variables. The distributions of study variables did not deviate from normality, and thus, parametric statistics were used. Differences and associations for the study variables with the demographic variables were assessed with *t*-tests, Pearson correlations, logistic regressions, z-ratios for the significance of the difference between two independent proportions, and the McNemar’s test for the significance of the difference between two correlated proportions. Logistic regression models were calculated for the influenza and COVID-19 vaccination, with background variables and the HBM and TRA variables. Generalized linear mixed models (GLMMs) were used to assess the significance of the differences in the contribution of the HBM and TRA variables to the odds of adherence to influenza vs. COVID-19 vaccination. Type of disease (influenza vs. COVID-19) was defined as the within-subjects variable.

## 3. Results

About 50% of the nurses adhered to influenza vaccination in 2020/2021, compared with about 62% of them in 2021/2022, a significant difference (McNemar’s *p* < 0.001) (Table 2). All in all, about 45% of the nurses adhered to influenza vaccination in both years. About two-thirds of the sample reported being fully vaccinated for COVID-19, a significantly higher percentage than those being twice vaccinated for influenza (McNemar’s *p* < 0.001).

Vaccination for COVID-19 was more common than for influenza (Table 3). Accordingly, perceived susceptibility and severity and attitudes supporting vaccination were higher for COVID than for influenza. No differences were found regarding perceived benefits or barriers. Significant correlations were found among the variables, regarding both influenza and COVID-19. Vaccination was positively associated with perceived susceptibility and severity, perceived benefits, and supporting attitudes and negatively associated with the barriers, regarding both types of vaccinations (except for perceived susceptibility and severity concerning COVID-19). Positive associations were found between perceived susceptibility and severity, perceived benefits, and supporting attitudes, and all were negatively associated with the barriers, regarding both types of vaccinations (except for perceived susceptibility and severity with the barriers). It should be noted as well that positive and high associations were found between the same HBM and TRA variables, regarding influenza and COVID-19 (*r* = 0.62 to *r* = 0.70, *p* < 0.001, for benefits with benefits, barriers with barriers, etc.).

Several associations with background variables were found to be significant. Influenza vaccination was more frequent among Jewish (*n* = 62; 54.4%) than non-Jewish (*n* = 114; 41.9%) nurses (*Z* = 2.25; *p* = 0.025). Likewise, COVID-19 vaccination was more frequent among Jewish (*n* = 90; 78.9%) than non-Jewish (*n* = 174; 64.0%) nurses (*Z* = 2.89; *p* = 0.004). Further, the odds of vaccination for COVID-19 were higher with older age (*OR* = 1.04; 95% *CI* = 1.02; 1.07; *p* < 0.001) and were thus higher with more years in nursing (age and years in nursing: *r* = 0.89; *p* < 0.001).

In addition, perceived susceptibility and severity, regarding both types of vaccinations, was higher among female nurses (influenza *M* = 3.3 *SD* = 0.9, COVID *M* = 3.6 *SD* = 0.8) than among male nurses (influenza *M* = 3.0 *SD* = 0.9, COVID *M* = 3.3 *SD* = 0.8) (influenza: *t*(384) = 2.63, *p* = 0.009, COVID: *t*(384) = 3.43, *p* > 0.001).

Perceived benefits, regarding both types of vaccinations, were positively related to age (influenza *r* = 0.12, *p* = 0.016, COVID *r* = 0.19, *p* < 0.001). They were higher among Jewish nurses (influenza *M* = 3.6 *SD* = 1.3, COVID *M* = 3.6 *SD* = 1.2) than among non-Jewish ones (influenza *M* = 3.2 *SD* = 1.2, COVID *M* = 3.2 *SD* = 1.1) (influenza: *t*(190.46) = 2.73, *p* = 0.007, COVID: *t*(384) = 2.81, *p* = 0.005). Quite similarly, the perceived barriers were negatively related to age (influenza *r* = −0.13, *p* = 0.012, COVID *r* = −0.20, *p* < 0.001). They were higher among non-Jewish nurses (influenza *M* = 2.8 *SD* = 0.8, COVID *M* = 2.9 *SD* = 0.7) than among Jewish ones (influenza *M* = 2.4 *SD* = 0.9, COVID *M* = 2.3 *SD* = 0.8) (influenza: *t*(384) = 5.00, *p* < 0.001, COVID: *t*(178.81) = 5.99, *p* < 0.001). Other differences and associations were not found to be meaningful. Thus, further analyses were calculated while controlling for age, gender (1—male, 0—female), and ethnicity (1—Jewish, 0—non-Jewish).

Logistic regression models were calculated for the influenza and COVID-19 vaccination, with background variables and HBM and TRA variables (Table 4). The model for influenza vaccination with influenza-related variables was found to be significant, with about 33% of the variance being explained in it. Higher perceived susceptibility and severity, lower perceived barriers, and more supportive attitudes were related to a greater likelihood of adherence to influenza vaccination. The model for COVID-19 vaccination with COVID-19-related variables was found to be significant as well, with about 15% of the variance being explained in it. Beyond older age, higher perceived benefits were related to a greater likelihood of adherence to COVID-19 vaccination.

In addition, an attempt was made to assess the associations between the COVID-related variables and adherence to influenza vaccination. The model was found to be significant, with about 15% of the variance being explained in it. Higher perceived susceptibility and severity regarding COVID, lower perceived barriers regarding COVID vaccination, and more supportive attitudes toward COVID vaccination were related to a greater likelihood of adherence to influenza vaccination.

As shown in Table 4, perceived susceptibility and severity, perceived barriers, and attitudes were significant regarding influenza vaccination, while perceived benefits were significant regarding COVID-19 vaccination. These differences were assessed with generalized linear mixed models for type of vaccination by the HBM and TRA variables. Significant differences between the two models were found for the contribution of perceived susceptibility and severity (*OR* = 2.04, 95% *CI* = 1.49, 2.78, *p* < 0.001), perceived benefits (*OR* = 1.49, 95% *CI* = 1.06, 2.04, *p* = 0.020), and attitudes (*OR* = 1.79, 95% *CI* = 1.28, 2.44, *p* < 0.001). The difference in the perceived barriers was not significant (*OR* = 1.20, 95% *CI* = 0.89, 1.64, *p* = 0.233). That is, the contributions of perceived susceptibility and severity and attitudes toward influenza vaccination were significantly greater than their contributions toward COVID-19 vaccination, while the contribution of the perceived benefits of COVID-19 vaccination was significantly greater than their contribution toward influenza vaccination.

## 4. Discussion

Influenza and COVID-19 are respiratory viral diseases that pose a risk to healthcare workers. The main reasons given by HCWs for deciding to get vaccinated against these viruses are the need to protect themselves and the ethical commitment to avoid missing work days, especially during periods when the public is particularly in need of medical treatment and hospitalization. Vaccination is considered an effective approach to maintaining the health and disease resistance of the healthcare workforce [23].

The findings of the current study rely on nurses’ self-reported responses to a questionnaire about vaccinations during the winter seasons of 2020/2021 and 2021/2022, when waves of COVID-19 were still spreading across the country. Due to the difficulties in eradicating the COVID-19 virus and its potential threat to the health of the population, vaccination was made a national priority.

In Israel, COVID-19 was publicly perceived as more dangerous than influenza. Hence, the rate of influenza vaccination was lower than the rate of COVID-19 vaccination. During 2021, similar coverage was reported in Australia [24].

In the current study, participating nurses reported a higher rate of vaccination in the winter of 2021/2022 (61.9%) than in the previous winter of 2020/2021 (49.5%). The Ministry of Health (MOH) reported different results, showing a 30% decrease in compliance with seasonal influenza vaccination among HCWs during the winter 2021–2022 compared to previous years. This difference between the current study’s findings and those reported by the MOH stems from the fact that this study included 386 participants, while the MOH data were collected nationwide at all medical centers. Moreover, the MOH files included all administered vaccinations, whereas this study relied on self-reporting, which may have been biased. In addition, the MOH statistics cover the autumn and winter season, which is different from the time covered by the present study (March-April 2022).

There are several plausible explanations for the MOH findings showing a decline in influenza vaccinations among nurses: Nurses may have experienced vaccination fatigue due to the need for repeated COVID-19 vaccine boosters. In addition, the health authorities directed their attention toward encouraging COVID-19 vaccination due to the critical threat to the public, paying much less attention to the influenza vaccine campaign [1]. Moreover, when COVID-19 era restrictions such as face masks and social distancing were lifted in the winter of 2021–2022, influenza and COVID-19 morbidity in Israel increased. Hence, medical centers were overloaded with patients infected with both viruses and lacked sufficient manpower due to isolations and morbidity among HCWs.

A study conducted in Poland surveyed HCWs regarding their opinions on vaccine hesitancy and their attitudes toward the COVID-19 and influenza vaccinations [25]. The participants included nurses, physicians, medical students, and other health professionals. That study was conducted during the autumn of 2020, before the COVID-19 vaccine was available. Thus, it examined respondents’ willingness to receive the influenza vaccine (61.1%) and their intention to get vaccinated for COVID-19 (68.7%). Ronn et al. [26] reported that in 2022, among adults in the US, influenza vaccination coverage was lower than COVID-19 vaccination coverage.

The current study’s findings point to an association between COVID-19 vaccination and the likelihood of receiving influenza vaccination among nurses in Israel. A similar outcome was reported by Andrejko et al. [27], who studied the receipt of seasonal vaccines for influenza and COVID-19 during 2021–2022 among the general public in California. During 2020–2022, the period of the COVID-19 pandemic, influenza virus vaccine receipt worldwide was lower than in previous years. Their results showed that those who received the COVID-19 vaccine were more likely to receive the influenza vaccine.

The outcomes of the current study regarding perceived benefits were related to age for both influenza and COVID-19 vaccinations, whereas perceived barriers were negatively related to age. The findings indicated that nurses’ age was associated with higher odds of complying with COVID-19 vaccination. Likewise, Wiysonge et al. [28] investigated the compliance with COVID-19 vaccination among HCWs in South Africa. Their findings indicated that older participants were more likely to comply with vaccination than younger participants. Kwok et al. [29] reported that older age was associated with higher influenza vaccine receipt among nurses in Hong Kong, China (49% compliance rate). At that time, however, only the intention to receive the COVID-19 vaccine could be surveyed [29]. Bellali et al. [30] also found that increased age was a predictive factor for influenza vaccination among HCWs in Greece. In contrast, other studies in Italy revealed that the HCW response to influenza vaccination was linked to younger age [3,5].

Gender was a statistically significant factor in the present study in determining vaccination receipt for both influenza and COVID-19, with female nurses in Israel exhibiting higher vaccination rates. In contrast, Dettori et al. [3] examined cohort study records (2018–2021) at an Italian University hospital and found generally higher influenza vaccination coverage among male HCWs than among female HCWs (*p* = 0.0063). In a study covering twelve countries, Parisi et al. [31] reported that neither age nor gender affected vaccine compliance or vaccine hesitancy among HCWs.

To the best of our knowledge, this is the first study to examine both influenza and COVID-19 vaccination among nurses in Israel. The current study analyzed the Health Belief Model (perceived susceptibility, severity, and benefits of the vaccines) together with the Theory of Reasoned Action (attitudes and social influences) among nurses who were vaccinated against influenza and COVID-19, as well as the interaction between these two models.

The HBM was developed to explain sociopsychological aspects of health-related behaviors and was used as a tool to assess factors that may influence compliance with influenza and COVID-19 vaccination among HCWs. The current study demonstrated that among nurses in Israel, HBM variables were significant in the case of influenza vaccination. Silva et al. [32] reviewed studies using HBM to describe the reasons for influenza vaccine receipt reported by HCWs. The study found that influenza vaccination compliance was related to susceptibility, severity, benefits, and barriers, supporting the present research showing a correlation between HBM variables and influenza vaccination. HCWs in high-risk wards in a hospital in West China were surveyed (November 2022) to examine influenza vaccination hesitancy. That investigation revealed that perceived susceptibility, perceived benefits, and perceived barriers were significantly correlated to vaccine compliance [33]. In 2020, Alhalaseh et al. conducted a study in Jordan using HBM variables to predict HCWs’ intentions to receive the influenza vaccine. Perceived benefits were found to be a significant predictor of vaccination intention [34].

The current study also found that perceived benefits were a significant variable contributing to COVID-19 vaccination compliance. Other studies are in agreement with the current result. In 2024, Youssef et al. [35] surveyed COVID-19 vaccine compliance among HCWs in Lebanon. The findings showed that willingness to be vaccinated was associated with factors such as susceptibility, perceived benefits, and recent influenza vaccination, with 65.8% expressing the intention to be vaccinated for COVID-19. Wiysonge et al. found that perceived benefits were a predictive factor in COVID-19 vaccine compliance among HCWs [28].

Getachew et al. [36] used the HBM model to assess factors associated with COVID-19 vaccine compliance among HCWs in Ethiopia. Perceived disease susceptibility and severity as well as age were significantly related to willingness to receive the COVID-19 vaccine. Their results are in line with the present study (Table 3).

Mbele et al. [37] analyzed COVID-19 vaccination receipt among HCWs in Ghana and found that a positive attitude was a predictive factor. The present study calculated models indicating that compliance with influenza vaccination may be explained by individuals’ decisions regarding COVID-19, which are influenced by high perceived susceptibility and severity of COVID-19 along with low perceived barriers to COVID-19 vaccination. Supportive attitudes toward COVID-19 vaccination also played a role in determining influenza vaccination compliance.

Several studies investigated the associations between the administration of influenza and COVID-19 vaccinations in the same season. Sani et al. [4] explored the attitudes of HCWs in Italy regarding influenza vaccination during the COVID-19 pandemic. The results of this study’s online questionnaire (July to October 2020) showed that the COVID-19 era significantly augmented HCWs’ attitudes toward influenza vaccination.

Parisi et al. [31] studied HCWs’ hesitancy regarding COVID-19 and influenza vaccination in twelve countries from October 2022 to April 2023. The results of this web-based survey of 7793 HCWs indicated that influenza vaccination behavior was related to COVID-19 vaccine hesitancy, along with other factors such as fear of COVID-19 and information sources. A shift toward increased vaccination against influenza was shown to be positively influenced by experience with COVID-19. Attitudes toward vaccination were not influenced by the COVID-19 pandemic. The most common reasons for COVID-19 vaccination compliance included social responsibility toward high-risk populations, a work environment marked by a high risk of contracting COVID-19, self-protection and protection of family members, and belief in the preventive effectiveness of the COVID-19 vaccine. Negative attitudes toward vaccination among HCWs contributed to low odds of compliance.

A multicenter survey of three Italian hospitals conducted in 2020 [4] evaluated HCWs’ attitudes toward influenza vaccination. This study showed an increase in awareness of the vaccine, which could be explained by the ongoing COVID-19 pandemic at that time. Yet, the influenza vaccination rate (53.4%) could be further improved. The study was conducted before the COVID-19 vaccine was launched, and 35.2% of responding HCWs expressed their intention to get vaccinated when the COVID-19 vaccine became available [4].

Different outcomes were obtained by Koh et al. [38] in a study that aimed to identify factors affecting COVID-19 vaccine compliance and hesitancy among HCWs in Singapore. Analysis of an online survey conducted in 2021 showed that COVID-19 compliance was not affected by previous influenza vaccination, self-perceived risk (HBM), age, or gender.

A survey conducted in 2021 among HCWs in Cape Town, South Africa, sought to assess COVID-19 vaccine compliance upon its initial roll-out [28]. The research outcomes indicated that factors predicting vaccine compliance were related to the altruistic nature of HCWs, such as the willingness to protect others and considering COVID-19 vaccination as a collective action tool for disease control.

Della Polla [5] examined HCWs at five hospitals in southern Italy. Participants answered a questionnaire that focused on attitudes (perceived risks of seasonal influenza to personal health and risk of passing on the influenza virus to patients) and reasons for vaccination. The participating HCWs demonstrated remarkable responsibility, with 75.4% believing that an infected HCW can transmit the virus to a patient. This study demonstrated that attitudes are valuable in an individual’s decision (e.g., the perceived severity of influenza was lower than the perceived risk of passing the influenza virus to patients and the risk of infection).

Belinghehri et al. [9] also examined the association between influenza vaccination and the risk of contracting COVID-19 among HCWs at a hospital in the Lombardy region in northern Italy. The results found no relationship between COVID-19 (diagnosed either by serology testing to detect IgG antibodies or by PCR diagnosis on swab samples) and getting the influenza vaccine. This study further supports the recommendation that HCWs should comply with influenza vaccination as a measure to ease the burden of hospitalizations and decrease symptom severity.

In a 2021 systematic review and meta-analysis, Gualano et al. [6] assessed HCWs’ attitudes toward a mandatory influenza vaccination policy between 2003 and 2019. The findings indicated that 40% of nurses would be willing to comply with influenza vaccination as a means to reduce the impact of influenza infection. Agreement with the mandatory vaccination policy among vaccinated HCWs was significantly higher than among non-vaccinated HCWs (RR: 1.94; *95% CI*: 1.48–2.55, *p* < 0.01). Moreover, Parisi found that the predictors of vaccine acceptance included greater fear of contracting COVID-19 infection and higher perceived severity of the disease [31].

A civilized society takes care of elderly people and young children, who are fragile or have weaker immune systems than healthy adults. Mandatory influenza vaccination, whether proposed or imposed, seeks to protect vulnerable populations who, by virtue of their condition, tend to visit HCW settings frequently. Because HCWs are exposed to numerous patients during their shifts, efforts are necessary to ensure that they are not a source of contagion but rather are able to take care of those who are sick. Patient safety as well as missed days of work were mentioned among the main reasons for HCWs’ responsiveness to influenza vaccination [6].

This study has several limitations. One is that that COVID-19 vaccination was not mandatory for HCWs in Israel. Parisi et al. [31] reported that in 2025, COVID-19 vaccination was mandatory for HCWs in Australia, Canada, France, Italy, and the USA. Israel does not implement a standardized “vaccinate or mask” policy for healthcare workers. There is no legal mandate requiring healthcare workers to be vaccinated against influenza or COVID-19, with emphasis placed on encouraging rather than enforcing vaccination. During the peak of the COVID-19 pandemic, universal masking was temporarily required, but current policies are more flexible and institution-dependent. In the Israeli context, vaccination decisions are framed primarily as professional responsibilities rather than institutional requirements with workplace consequences. This cultural and policy framework likely influences questionnaire responses, as participants may base their decisions on personal and professional considerations rather than potential workplace restrictions. Another limitation is that participants answered the survey via the internet. Thus, the current study may unintentionally be biased to exclude people who are less active on social networks. The biased dissemination of the snowball sampling method also poses a limitation, though on the other hand, participants were randomly selected. The self-reporting method may also be biased as it poses a challenge to individuals’ reliability. For example, answering the questionnaire may have an impact on the number of vaccine shots participants get. Finally, the decision to be vaccinated may be influenced by factors other than those examined in the questionnaire.

Future research directions could include medical center-based surveys disseminated via internal workplace email. In addition, the reliability of self-reports regarding the number of vaccinations per participant could be verified through medical identification records. Moreover, it would be interesting to examine the responses of nurses stratified according to type of workplace, e.g., medical center intensive care ward, geriatric or neonatal intensive care unit, or community medical clinic.

## 5. Conclusions

Hesitancy regarding influenza vaccination should be further explored to determine additional factors affecting nurses’ opinions regarding barriers. To increase vaccination compliance, policymakers should give priority to widespread campaigns promoting vaccination as protection against viral infections. Medical education is of paramount importance in convincing HCWs of the proven necessity of getting vaccinated and of collectively sharing responsibility for community/public health.

The present research is important and unique in that it provides insight about factors related to responses to different vaccines during a pandemic. The strength of the current study lies in its simultaneous examination of the compliance with both vaccinations (influenza and COVID-19) and its analysis of factors influencing an individual’s decision to receive both types. The current study also has implications for preventing the global spread of other vaccine-preventable contagious maladies through efforts to reduce vaccine hesitancy. Training programs are recommended to promote vaccinations, in addition to advocacy for combined COVID-19 and influenza vaccination each year.

## Figures and Tables

**Table 1:** Participants’ sociodemographic characteristics (*N* = 386).

Characteristics	
Age, mean years (*SD*) ^1^, range	37.1 (11.0), 22–62
Gender, *n* (%)	
Male	103 (26.7)
Female	283 (73.3)
Marital status *n* (%)	
Single	81 (21.0)
Married, in a relationship	287 (74.3)
Divorced, widowed	18 (4.7)
Children, yes, *n* (%)	265 (68.7)
Mean number of children (*SD*) ^1^, range (*n* = 265)	2.5 (1.1), 1–8
Religion, *n* (%)	
Jewish	114 (29.5)
Muslim	186 (48.2)
Christian	72 (18.7)
Druze	14 (3.6)
Religiosity, *n* (%)	
Secular	142 (36.8)
Partly religious	155 (40.1)
Religious	89 (23.1)
Level of education, *n* (%)	
Practical nurse	16 (4.1)
Certified nurse	96 (24.9)
Certified nurse with B.S.N. ^1^	115 (29.8)
Certified nurse with B.S.N. ^1^ and professional course	103 (26.7)
M.S.N. ^1^	56 (14.5)
Years in nursing, mean years (*SD*) ^1^, range	11.6 (11.0), 0.5–41
Work place, *n* (%)	
Hospital	235 (60.9)
Community health service	69 (17.9)
Nursing home	82 (21.2)
Health, *n* (%)	
Excellent	128 (33.2)
Very good	153 (39.6)
Good	85 (22.0)
Reasonable, not good	20 (5.2)
Chronic illness, yes, *n* (%)	71 (18.4)
Permanent medication, yes, *n* (%)	76 (19.7)

^1^ Abbreviations: *SD*, Standard Deviation; B.S.N., Bachelor of Science in Nursing; M.S.N., Master of Science in Nursing.

**Table 2:** Participants’ vaccination (*N* = 386).

Characteristics	*n* (%)
Influenza vaccination 2020/2021, *n* (%)	191 (49.5)
Influenza vaccination 2021/2022, *n* (%)	239 (61.9)
Influenza vaccination in both years, *n* (%)	176 (45.6)
COVID-19 ^1^ vaccination, *n* (%)	264 (68.4)

^1^ Abbreviation: COVID-19, Coronavirus disease 2019.

**Table 3:** Means, standard deviations, and correlations for the study variables (*N* = 386).

	Influenza *M* (*SD*) *	COVID-19 *M (SD)*	*t*(385) (*p*)	1.	2.	3.	4.	5.
1. Vaccination (yes)	0.5 (0.5)	0.7 (0.5)	McNemar’s *p* < 0.001	1	0.39 ***	0.43 ***	−0.25 ***	0.42 ***
2. Perceived susceptibility and severity	3.2 (0.9)	3.3 (0.8)	8.78 (*p* < 0.001)	0.07	1	0.56 ***	−0.09 ***	0.53 ***
3. Perceived benefits	3.3 (1.2)	3.3 (1.2)	1.15 (*p* = 0.252)	0.28 ***	0.48 ***	1	−0.32 ***	0.70 ***
4. Perceived barriers	2.7 (0.9)	2.7 (0.8)	0.84 (*p* = 0.402)	−0.16 **	−0.02	−0.030 ***	1	−0.18 ***
5. Attitudes	2.6 (1.1)	2.9 (1.2)	6.94 (*p* < 0.001)	0.18 ***	0.44 ***	0.70 ***	−0.17 **	1

* *p* < 0.05, ** *p* < 0.01, *** *p* < 0.001. Note. Above diagonal—correlations for influenza vaccination; below diagonal—correlations for COVID-19 vaccination. Range 1–5.

**Table 4:** Logistic regression models for influenza and COVID-19 vaccination, with HBM and TRA variables (*N* = 386).

Variable	Influenza Vaccination with Influenza Variables *OR* ^1^ (95% *CI* ^1^) (*p*)	COVID ^1^-19 Vaccination with COVID-19 Variables *OR* ^1^ (95% *CI* ^1^) (*p*)	Influenza Vaccination with COVID-19 Variables *OR* ^1^ (95% *CI* ^1^) (*p*)
Age	0.99 (0.97, 1.02) (*p* = 0.656)	1.03 (1.01, 1.06) (*p* = 0.017)	0.99 (0.97, 1.02) (*p* = 0.728)
Gender (male)	1.04 (0.60, 1.79) (*p* = 0.887)	0.94 (0.55, 1.58) (*p* = 0.805)	1.07 (0.64, 1.77) (*p* = 0.804)
Ethnicity (Jewish)	1.11 (0.60, 2.06) (*p* = 0.728)	1.26 (0.68, 2.35) (*p* = 0.465)	1.36 (0.78, 2.38) (*p* = 0.277)
Perceived susceptibility and severity	1.81 (1.30, 2.53) (*p* < 0.001)	1.14 (0.87, 1.48) (*p* = 0.342)	1.58 (1.17, 2.14) (*p* = 0.003)
Perceived benefits	1.27 (0.95, 1.70) (*p* = 0.107)	1.67 (1.24, 2.24) (*p* < 0.001)	1.23 (0.98, 1.53) (*p* = 0.067)
Perceived barriers	0.62 (0.46, 0.85) (*p* = 0.002)	0.87 (0.63, 1.21) (*p* = 0.412)	0.71 (0.52, 0.97) (*p* = 0.030)
Attitudes	1.56 (1.16, 2.10) (*p* = 0.003)	1.02 (0.78, 1.34) (*p* = 0.882)	1.39 (1.08, 1.80) (*p* = 0.010)
Nagelkerke’s *R^2^*	0.33, *p* <0.001	0.15, *p* <0.001	0.15, *p* < 0.0001

^1^ Abbreviations: OR, odds ratio; *CI*, confidence interval; COVID-19, Coronavirus disease 2019.

## Data Availability

The raw data supporting the conclusions of this paper will be made available by the author on request.

## Abbreviations

CI	Confidence interval
COVID-19	Coronavirus disease 2019
HBM	Health Belief Model
OD	Odds ratio
SD	Standard deviation
TRA	Theory of Reasoned Action
WHO	World Health Organization
